# Molecular Epidemiology of Oropouche Virus, Ceará State, Brazil, 2024

**DOI:** 10.3201/eid3104.241471

**Published:** 2025-04

**Authors:** Shirlene T.S. de Lima, Xinyi Hua, Ingra M. Claro, Carlos Garcia Filho, Leda M. Simões Mello, Ronaldo de Jesus, Amanda Bleichrodt, Ana Maria P.C. Maia, Ana Carolina B.M. Máximo, Karene F. Cavalcante, Antônio Carlos L. Firmino, Larissa M.F. Duarte, Luiz Osvaldo R. da Silva, Andre R.R. Freitas, Isaac Chun-Hai Fung, Gerardo Chowell, Pritesh Lalwani, Luciano P.G. Cavalcanti, Camila M. Romano, José Luiz Proenca-Modena, William M. de Souza

**Affiliations:** University of Kentucky, Lexington, Kentucky, USA (S.T.S. de Lima, X. Hua, I.M. Claro, W.M. de Souza); Laboratório Central de Saúde Pública do Ceará, Fortaleza, Brazil (S.T.S. de Lima, L.M. Simões Mello, A.C.B.M. Máximo, K.F. Cavalcante, A.C.L. Firmino, L.M.F. Duarte); Universidade Estadual de Campinas, Campinas, Brazil (S.T.S. de Lima, J.L. Proenca-Modena); Universidade de Fortaleza, Fortaleza (C.G. Filho); Universidade Federal de Minas Gerais, Belo Horizonte, Brazil (R. de Jesus, L.O.R. da Silva); Georgia State University, Atlanta, Georgia, USA (A. Bleichrodt, G. Chowell, L.P.G. Cavalcanti); Universidade Federal do Ceará, Fortaleza (A.M.P.C. Maia); Faculdade São Leopoldo Mandic, São Paulo, Brazil (A.R.R. Freitas); Georgia Southern University, Statesboro, Georgia, USA (I.C.-H. Fung); Fiocruz Amazônia, Manaus, Brazil (P. Lalwani); University of São Paulo, São Paulo (C.M. Romano)

**Keywords:** viruses, vector-borne infections, arbovirus, Oropouche virus, neglected tropical diseases, Oropouche fever, vector-borne orthobunyavirus, Peribunyaviridae, Brazil

## Abstract

During May–December 2024, we detected Oropouche virus (OROV) in 13.9% (263/1,890) of febrile patients in Ceará state, Brazil. Genomic sequencing revealed those cases were caused by a novel OROV reassortant previously identified in the Amazon region. Our data show the introduction and establishment of OROV transmission in Ceará, northeastern Brazil.

Oropouche virus (OROV) is a neglected vectorborne orthobunyavirus that has caused Oropouche fever in the Amazon region since the 1950s (*1*,*2*). OROV infection usually causes febrile illness but can also lead to neurologic diseases, pregnancy complications, and death (*1*,*3*–*7*). OROV is primarily transmitted to humans by *Culicoides paraenesis* midges in the human-amplified and enzootic cycles, and pale-throated sloths are potential amplifier hosts (*1*,*8*). As of March 2025, no specific antiviral drugs or vaccines were available to treat or prevent Oropouche fever.

Oropouche fever burden remains undetermined, but some studies have estimated >500,000 cases in the Amazon Basin since the 1950s (*1*). In November 2023, a substantial increase in the incidence of Oropouche fever was observed in the Amazon region in Brazil, Bolivia, Peru, and Colombia (*4*,*9*). The reemergence has been linked with the novel OROV reassortant identified in the Amazon Basin and later spread to previously nonendemic areas (*9*–*11*). However, knowledge about introducing and establishing OROV outside the Amazon region remains limited. We conducted a molecular epidemiology study to investigate the active circulation of OROV in patients with acute febrile illness in 2024 in Ceará state, Brazil.

## The Study

During January 1–December 28, 2024 (epidemiologic weeks 1–52), we collected serum samples from patients with acute febrile illness at primary healthcare units in Ceará state, Brazil. OROV and Mayaro virus laboratory diagnosis has been included for all persons in Ceará since April 3, 2024. OROV testing includes all physician-suspected cases, plus 10% of negative dengue samples. On August 9, 2024, all negative samples for dengue virus (DENV), chikungunya virus (CHIKV), and Zika virus (ZIKV) were also tested for OROV. We also conducted retrospective OROV testing in samples from patients with febrile illnesses during January 3–April 3, 2024. We obtained patient demographic information, sample collection date, symptom onset date, and detailed symptoms from the Brazilian Laboratorial Environment Management System. RNA was extracted from serum samples and subjected to real-time reverse transcription PCR to detect RNA of OROV, Mayaro virus, CHIKV, ZIKV, and DENV. Next, we sequenced OROV-positive samples using the Illumina platform (https://www.illumina.com) and conducted phylogenetic and reassortment analyses (Appendix). In addition, we performed epidemiologic time series, mapping, age–sex distribution, and statistical hypothesis tests (Appendix).

**Figure 1:**
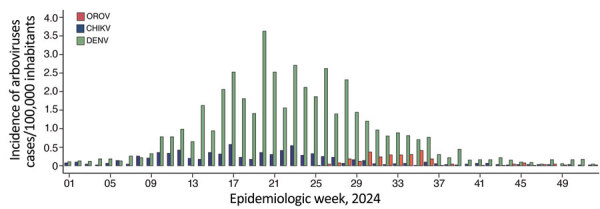
Incidence of laboratory-confirmed Oropouche fever, dengue, and chikungunya cases in study of molecular epidemiology of OROV, Ceará state, Brazil, January–December 2024. Incidence is shown per epidemiologic week at state-level from epidemiologic week 1 (December 31, 2023–January 6, 2024) to epidemiologic week 52 of 2024 (December 22–28, 2024). CHIKV, chikungunya virus; DENV, dengue virus; OROV, Oropouche virus.

**Figure 2:**
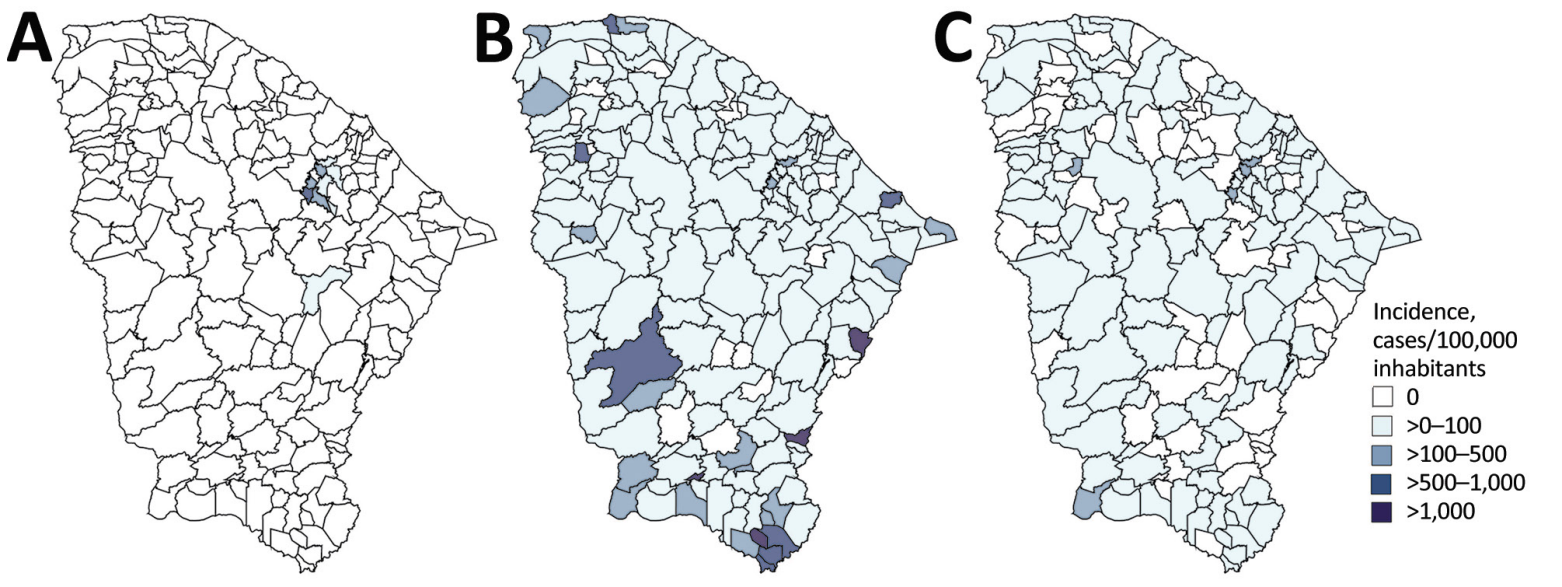
Geographic incidence of laboratory-confirmed Oropouche fever (A), dengue (B), and chikungunya virus (C), by municipality level, in study of molecular epidemiology of Oropouche virus, Ceará state, Brazil, January–December 2024.

Of 1,890 patients tested by real-time reverse transcription PCR, 13.9% (n = 263) were positive for OROV, representing a state-level incidence of ≈3 cases/100,000 inhabitants. No persons with Oropouche fever in Ceará had travel history to the Amazon region. The first detected Oropouche fever case occurred on May 20, 2024, at epidemiologic week 21 (Figure 1). Oropouche fever cases were detected in 8 municipalities in Ceará state: Aratuba, Banabuiú, Baturité, Capistrano, Mulungu, Pacoti, Palmácia, and Redenção. The municipality-level cumulative incidence of Oropouche fever across those municipalities varied from 5.7 to 602.1 cases/100,000 inhabitants. Capistrano (26.6%, 70/263) and Aratuba (26.2%, 69/263) reported the highest number of cases in Ceará (Figure 2).

**Figure 3:**
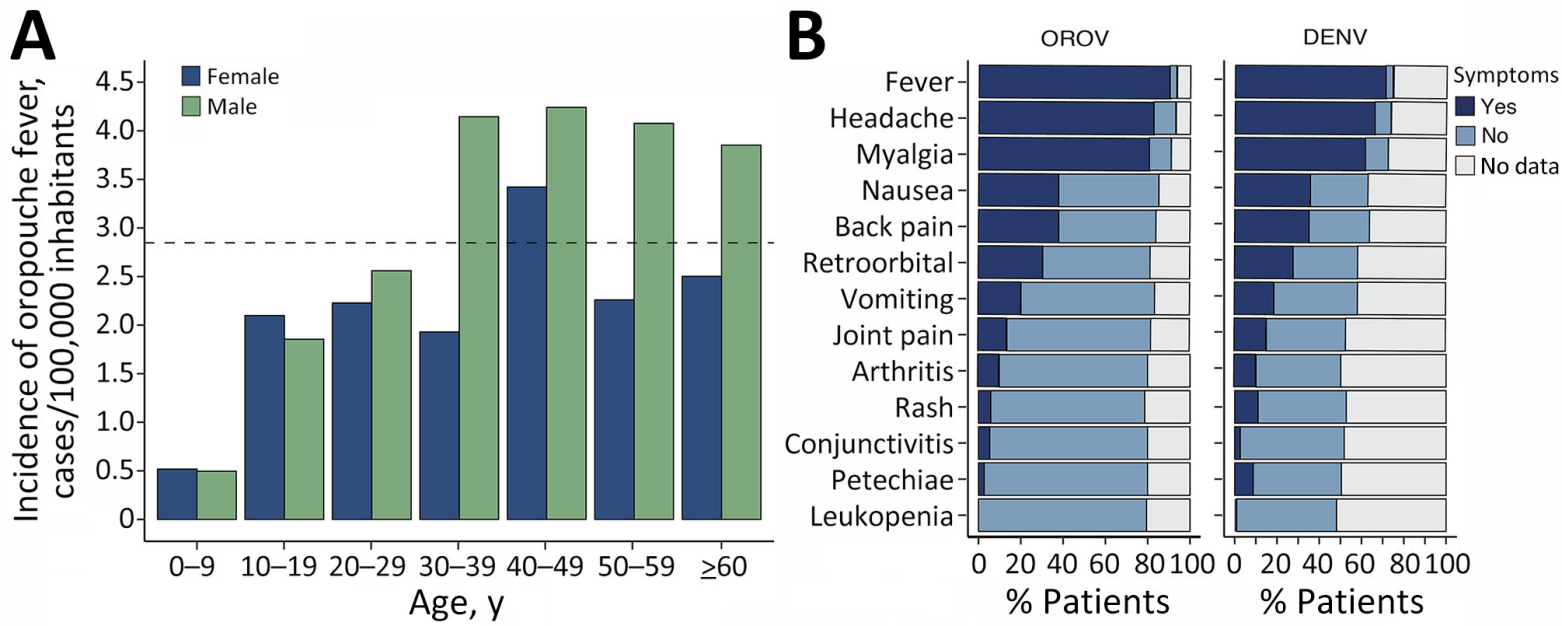
Age–sex structure and clinical features of Oropouche fever cases in study of molecular epidemiology of OROV, Ceará state, Brazil, January–December 2024. A) Oropouche fever incidence based on the age–sex distribution of laboratory-confirmed cases. Dashed line indicates the cumulative incidence of 2.85/100,000 population in Ceará state. B) Signs and symptoms of OROV (n = 224) and DENV (n = 307) confirmed by quantitative reverse transcription PCR. DENV, dengue virus; OROV, Oropouche virus.

Among Oropouche fever cases, median patient age was 40 years (interquartile range [IQR] 28–54 years), and the male-to-female ratio was 1.3. Persons 40–49 years of age had significantly higher incidence than those <9 years of age, and incidence was significantly higher among male than female patients (p<0.05) (Figure 2, panel A; Appendix Table 1). Median time between symptom onset and sample collection was 3 (IQR 1–3) days, and cycle threshold values were 18–38 (median 23, IQR 23–31). The most common symptoms reported by patients with Oropouche fever were fever (90.6%, 203/224), headache (83.0%, 186/224), and myalgia (80.1%, 181/224), similar to those observed in patients with dengue (Figure 3; Appendix Tables 2, 3).

**Figure 4:**
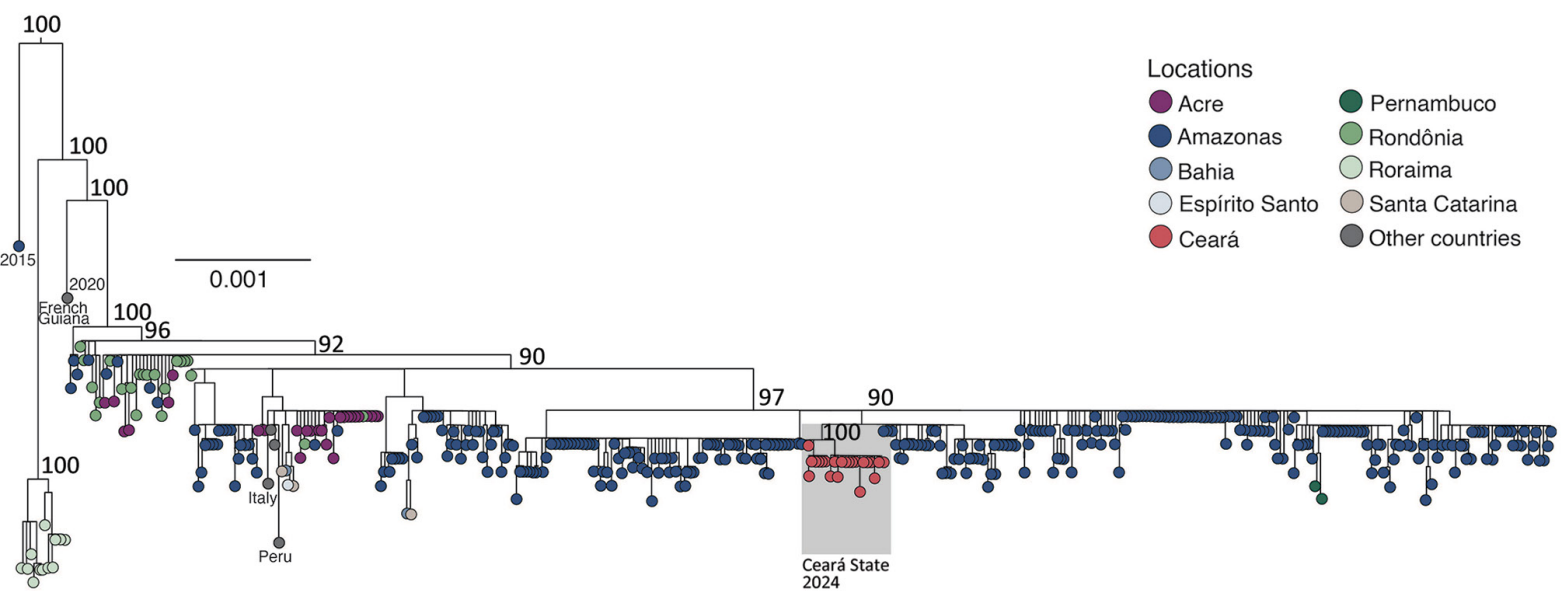
Phylogenetic analysis of the Oropouche virus (OROV) medium segment, 2023–2024 epidemic clade, in study of molecular epidemiology of OROV, Ceará state, Brazil, January–December 2024. The maximum-likelihood phylogenetic tree includes 420 representative OROV genomes from the 2023–2024 epidemic, with 22 newly sequenced genomes from Ceará (red dots highlighted in gray) generated in this study. Tip colors correspond to the state of origin for each sample. The phylogeny is midpoint-rooted for clarity. Scale bar represents the evolutionary distance in substitutions per nucleotide site. Bootstrap values, calculated from 1,000 replicates, are displayed at key nodes. GenBank accession numbers for all sequences used are described in the Appendix.

We sequenced near-complete coding sequences (>70% coverage across all 3 segments) for 22 OROV strains. Those sequences had an average depth of coverage of 4,725× for the large segment, 8,510× for the medium segment, and 2,980× for the small segment. We submitted the OROV sequences to GenBank (accession nos. PQ381540–605). Maximum-likelihood phylogenetic analyses revealed that all OROV strains from Ceará clustered within a highly supported monophyletic clade (bootstrap support 100%) in all 3 genomic segments within 2023–2024 strains (Figure 4; Appendix Figures 2, 3). Our analysis confirmed that OROV strains from Ceará resulted from the OROV reassortant described in the Amazon Basin during 2022–2023 (*9*). The amino acid identity analysis showed a similarity of >99.9% between OROV strains from Ceará and all 3 genomic segments of 420 reassortant OROV strains sampled from Amazonas, Acre, Rondônia, Roraima, Bahia, Pernambuco, Espírito Santo, and Santa Catarina states, as well as OROV strains reported in Peru, French Guiana, and Italy.

A total of 4,262 dengue cases and 740 CHIKV cases were detected in Ceará during January 1–December 28, 2024, which corresponds to cumulative incidences of 46.2/100,000 inhabitants for dengue and 8.0/100,000 inhabitants for CHIKV. Cases of DENV infection were detected in 90.8% (167/184) municipalities across Ceará, and the epidemic peak occurred between early April and late July (Figures 1, 2). Similarly, cases of CHIKV infection were identified in 67.9% (125/184) municipalities, for an incidence of <1 case/100,000 inhabitants per epidemiologic week during the same period (Figures 1, 2). All samples tested were negative for RNA of ZIKV and Mayaro virus.

## Conclusions

This study describes the introduction and establishment of OROV in Ceará state, northeastern Brazil. Our findings suggest a single introduction of OROV into Ceará, likely through a person infected in the Amazon region who traveled to Ceará during the viremic phase. We hypothesize this idea because of the similarity between the OROV strains of Ceará and those from Amazonas state, as well as their shared most recent common ancestor. Furthermore, OROV strains from Ceará are genetically related to other reassortant OROV strains that emerged in the Amazon region and spread to other areas in 2024 (*9*,*10*). This OROV reassortant has been associated with phenotypic changes that might contribute to increased virulence, enhanced viral fitness, and the ability to evade previous immunity (*11*).

As of December 28, 2024, OROV had been detected in only 8 rural municipalities in the Maciço de Baturité region of Ceará, in contrast to the widespread distribution of CHIKV and DENV across the state. Our findings support the hypothesis that the OROV emergence outside the Amazon region appears predominantly in small inner municipalities rather than in large urban centers, potentially because of the proximity to forest areas and local agricultural activities that might favor OROV transmission by midges (*12*). Conversely, DENV, CHIKV, and ZIKV are primarily transmitted by *Aedes aegypti* mosquitoes, which are well adapted to urban settings (*13*). Moreover, clinical symptoms and signs caused by Oropouche fever are similar to those observed in dengue patients, and laboratory diagnosis of OROV should be performed in patients with febrile illness in Ceará state.

The first limitation of our study is that it relied on healthcare-seeking behaviors, which could lead to an underestimate of the number of cases. Second, our focus was active OROV infections diagnosed using molecular methods, but further serologic studies are necessary to determine the population infected by OROV. Third, further entomologic investigations will be crucial for determining the OROV vector in Ceará and better mitigating future OROV outbreaks. In addition, molecular and serologic studies should be conducted to investigate the circulation of OROV in sloths within the Maciço de Baturité region to assess whether OROV has established an enzootic cycle in the area.

In summary, our study identified the introduction and establishment of OROV in Ceará state, Brazil. Our findings emphasize the importance of continuous surveillance to mitigate and interrupt the transmission of OROV in its early stages of establishment in Ceará and northeastern Brazil.

Appendix Additional information about molecular epidemiology of Oropouche virus, Ceará state, Brazil, 2024
